# Transbronchial lung cryobiopsy may be of value for nonresolving acute respiratory distress syndrome: case series and systematic literature review

**DOI:** 10.1186/s12890-020-01203-w

**Published:** 2020-06-29

**Authors:** Guowu Zhou, Yingying Feng, Shiyao Wang, Yi Zhang, Ye Tian, Xiaojing Wu, Ling Zhao, Dan Wang, Ying Li, Zheng Tian, Qingyuan Zhan

**Affiliations:** 1 Department of Pulmonary and Critical Care Medicine, Centre of Respiratory Medicine, China-Japan Friendship Hospital; National Clinical Research Centre for Respiratory Diseases, Institute of Respiratory Medicine, Chinese Academy of Medical Sciences, 2 Yinghuayuan East Street, Chaoyang District, Beijing, 100029 China; 2 Department of Pathology, China-Japan Friendship Hospital, Beijing, 100029 China

**Keywords:** TBLC, ARDS, Biopsy, Safety, Diagnostic yield

## Abstract

**Background:**

Identification of pathologic features is helpful for the management of nonresolving acute respiratory distress syndrome (ARDS). Transbronchial lung cryobiopsy (TBLC) is a novel biopsy technique that may have comparable utility to surgical biopsy. The aim of this study was to assess the value of TBLC in patients with nonresolving ARDS.

**Methods:**

All patients with nonresolving ARDS who underwent TBLC from January 2019 to August 2019 in a tertiary medical ICU were included. In addition, a literature search of TBLC for ARDS was performed by searching PubMed, EMBASE, ATS/ERS/APSR meeting abstracts, ClinicalTrials.gov, and Google Scholar. Data on complications, histologic diagnosis, management changes, and outcomes were analysed.

**Results:**

Five patients (three women and two men) underwent TBLC. None of the patients developed pneumothorax, although two patients developed massive bleeding, which was controlled by continuous occlusion using bronchial blockers. There were no procedure-related deaths. Diffuse alveolar damage (DAD) and alternative histologic patterns were found in two and three patients, respectively, resulting in management changes in all cases. The literature search yielded four studies, which together with the present study comprised data from 25 cases in which TBLC was used in nonresolving ARDS. The summary diagnostic yield was 92% (23/25). Only 44% (11/25) of cases were proven to be DAD. TBLC contributed to management changes in 80% of patients (20/25). Procedure-related complications consisted of pneumothorax (16%, 4/25), significant bleeding (12%, 3/25), and persistent air leaks (8%, 2/25). There were no procedure-related deaths. The follow-up survival rate was 61.9% (13/21).

**Conclusions:**

The complications of TBLC in selected patients with nonresolving ARDS may be acceptable. The procedure may have a high diagnostic yield and can lead to a re-evaluation of the diagnosis as well as changes in patient management. Further investigations with larger sample sizes are required.

## Background

Acute respiratory distress syndrome (ARDS) is common in critically ill patients. The majority of studies report a high rate of morbidity and a mortality rate in the range of 35–60% [1, 2]. The Berlin definition of ARDS takes into account clinical and radiological criteria in the diagnosis and classification without considering pathologic findings [3]. Diffuse alveolar damage (DAD) is a typical pathological finding in ARDS [4], but it is not seen in all ARDS patients nor is it specific to the disease. Studies have shown that only 43–45% of ARDS patients have DAD [5–8]. Identifying the pathologic pattern in nonresolving ARDS can facilitate treatment decision-making and improve outcomes [6, 8].

When the inciting event that results in ARDS is unclear or when an alternative diagnosis is under consideration, surgical lung biopsy (SLB) is one of the most common procedures to obtain a histologic diagnosis. Several studies have shown a positive impact of the histologic results of SLB in patients with nonresolving ARDS [6, 8]. However, SLB is performed in only 4–7% of patients with ARDS [6, 9] due to the high surgery risk and poor conditions of most patients who are candidates for the procedure. Moreover, the complication rate of SLB is approximately 30%, with complications including persistent air leak and bleeding [9]. The use of less invasive procedures, such as transbronchial lung biopsy (TBLB), in clinical practice has been limited due to difficulties in obtaining adequate tissue to provide confident pathologic results (the diagnostic yield is < 35%) [9, 10]. Transbronchial lung cryobiopsy (TBLC) is an alternative technique that has been widely used for the diagnosis of diffuse parenchymal lung disease (DPLD) [11]. The sample sizes are significantly larger than those obtained with TBLB, and the diagnostic yield approaches that of SLB with lower complication rates [12, 13]. These observations suggest that TBLC may be an alternative procedure to SLB in patients with nonresolving ARDS, with potential benefits including adequate tissue for identifying pathologies and less invasiveness. However, to the best of our knowledge, the use of TBLC in nonresolving ARDS has been examined in only a few case reports. Therefore, in this study we assessed the use of TBLC in nonresolving ARDS based on 25 cases from our institute and a literature review.

## Methods

### Patients

TBLC was conducted in the event of persistent respiratory failure after ongoing lung infection was ruled out by previous bronchoalveolar lavage (BAL) or when an alternative diagnosis was suspected based on the patient’s history, and clinical and radiologic presentation. The hospital records of all ARDS patients who underwent TBLC from January 2019 to August 2019 in our 26-bed tertiary medical ICU were reviewed. The inclusion criteria were as follows: age ≥ 18 years at the time of TBLC, findings consistent with the Berlin ARDS definition, and ARDS characterized as mild, moderate, or severe, as described in the Berlin definition, at the time of diagnosis and biopsy.

### TBLC procedure

**Fig. 1:**
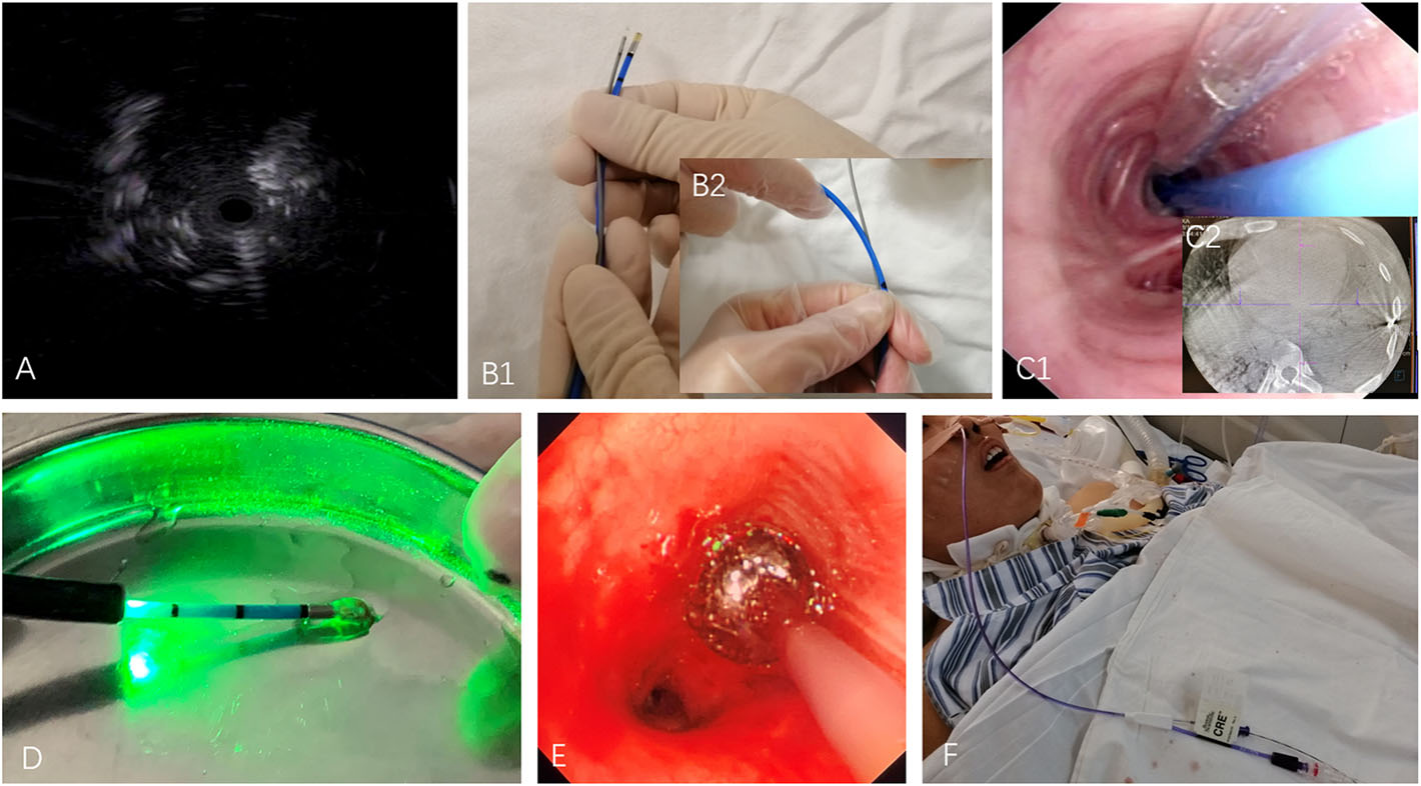
Radial probe endobronchial ultrasound (RP-EBUS) guided transbronchial lung cryobiopsy (TBLC) for acute respiratory distress syndrome (ARDS). **a** RP-EBUS screening of the target biopsy position. **b** Marking the biopsy distance on the cryoprobe compared to that on RP-EBUS. **c1** Prophylactic placement of the bronchial blocker and insertion of the cryoprobe into the target segment. **c2** Combined guidance with cone beam CT after placing the cryoprobe in patient 5. **d** Transbronchial lung cryobiopsy was performed after freezing for 4 s. **e** The bronchial blocker was filled to stop the bleeding. **f** Bronchial blockers were continuously placed in target bronchi for patients with massive bleeding

Five patients with ARDS underwent TBLC (Fig. 1). In four patients, the procedure was performed at the ICU bedside through an endotracheal tube (ETT), with the patient under deep sedation and supported by pressure control ventilation (F_i_O_2_ 100%, PEEP 0 cmH_2_O) or combined with extracorporeal membrane oxygenation (ECMO). The fifth patient received TBLC in a hybrid cone beam computed tomography (CBCT) operating room (OR). The procedure was conducted using rigid bronchoscopy with the patient under general anesthesia and ventilated by a high-frequency jet respirator (F_i_O_2_ 100%, respiratory rate 60 bpm, tidal volume 500 mL). In the four bedside procedures, bronchoscopy was first introduced through the nasal route to the lower airway after releasing the balloon on the ETT. Next, a long guidewire was inserted into the target lobe through the bronchoscopy working channel. Bronchoscopy was then pulled out alone and then reinserted through the ETT. Then, a bronchial blocker with a guide channel was introduced along the guidewire to the target lobe. Radial probe endobronchial ultrasound (RP-EBUS) (EU-ME1, Olympus, Tokyo, Japan) was used to identify the proper biopsy site (Fig. 1a). Under the guidance of preprocedurale CT images, the bronchoscope was advanced into the potential target bronchi as far as possible and then retracted 1–2 cm. When the surrounding RP-EBUS showed a heterogeneous echo without vascular presentation, the depth of the probe was marked. A 2.4 mm cryoprobe (ERBE, Solingen, Germany) was inserted to the same position (Fig. 1b, Fig. 1 C1). Cryobiopsy was performed (freeze time: 4 s) following probe positioning using carbon dioxide as the cryogen (Fig. 1d). After each biopsy, the bronchial balloon blocker (CRE balloon, Boston Scientific Microvasive, Natick, MA, USA) was immediately filled (0.5–1 atm) to stop the bleeding (Fig. 1e). Two to five biopsies were performed in each patient, and the sizes of the obtained samples were measured. For patients who underwent TBLC in the hybrid OR, flexible bronchoscopy and bronchial blockers were inserted through a rigid bronchoscope. Prior to TBLC, CBCT images (Artis Zee III ceiling, Siemens AG, Munich, Germany) were acquired to determine the exact position of the cryoprobe (Fig. 1 C2), which was placed under RP-EBUS guidance.

In patients with ECMO, the plan was to stop treatment with unfractionated heparin (UFH) 4 h before TBLC and then monitor the activated clotting time of whole blood (ACT). TBLC was performed when the ACT had decreased to within an acceptable range. After the procedure, the bronchial blocker was not removed until UFH had been reinitiated (Fig. 1f) and the ACT had recovered to the level previously achieved during ECMO with no active bleeding.

### Outcomes

Patients routinely underwent a postprocedure chest X-ray to screen for pneumothorax. Bleeding severity was graded on a scale of 4: no bleeding, mild bleeding (requiring suction for clearance but no other endoscopic procedures), moderate bleeding (requiring endoscopic procedures such as bronchial occlusion-collapse and/or instillation of ice-cold saline) and severe bleeding (causing haemodynamic or respiratory instability, requiring tamponade or other surgical interventions or transfusions) [14]. Other complications, if any, were recorded.

Data on the pathological diagnosis, treatment changes after TBLC and survival outcomes were obtained from the patients’ medical records.

### Literature search strategy

A literature search of PubMed, EMBASE, ATS meeting abstracts, ERS meeting abstracts, APSR meeting abstracts, ClinicalTrials.gov, and Google Scholar ending on November 25, 2019, was conducted using the following terms: “acute respiratory distress syndrome” or “ARDS,” and “cryobiopsy” or “cryoprobe,” without restrictions on language or publication year. The retrieved papers were read in their entirety to assess their appropriateness for this study of the value of TBLC in ARDS. Data on the characteristics of the TBLC procedure, related complications, pathological diagnosis, treatment changes and patient survival outcomes were extracted, and summary proportions were calculated based on individual cases.

## Results

### Patient characteristics

Five patients (three women and two men) with nonresolving ARDS underwent TBLC at our hospital. Their average age was 53 years (range: 31–68 years). The characteristics of these patients are summarized in Table 1. Four of them had underlying disease, including multiple sclerosis, nephrotic syndrome, impaired glucose tolerance and advanced lung adenocarcinoma. Four of the patients were diagnosed with severe ARDS and one with moderate ARDS according to the Berlin definition. All four patients with severe ARDS received mechanical ventilation (MV) support, including two patients who received MV in combination with veno-venous ECMO. UFH was used in patients with ECMO but was stopped 4 h before TBLC in one patient. In the other patient (patient 1), because flow-through ECMO decreased significantly after UFH was stopped, heparin use was continued until 30 min before the biopsy procedure. Respiratory support in patients with moderate ARDS consisted of high-flow nasal cannula oxygen therapy (HFNC).

**Table 1:** Clinical characteristics in nonresolving acute respiratory distress syndrome (ARDS) patients undergoing transbronchial lung cryobiopsy (TBLC)

Case	Age (years), gender	Underly diseases	ARDS severity	Ventilation settings	Guidance	Freezing time	Sample number and size	Complication	Finial pathology	Management changes	Outcome
1	39/F	MS	Severe	PCV (PC 14 cmH_2_O, F_i_O_2_ 90%, PEEP 11 cmH_2_O) and ECMO (4500 rpm, blood flow 6.3 L/min, gas flow 4 L/min, F_i_O_2_ 100%)	RP-EBUS	2.4 mm/4 s	2/20, 30 mm^2^	Severe bleeding	Fibrotic phase of DAD with underly infection	changes in antibiotic drugs and steroid discontinuation	Died
2	68/M	NS	Severe	PCV (PC 16 cmH_2_O, F_i_O_2_ 40%, PEEP 6 cmH_2_O) and ECMO (3120 rpm, blood flow 3.86 L/min, gas flow 3.5 L/min, F_i_O_2_ 100%)	RP-EBUS	2.4 mm/4 s	3/12, 20, 35 mm^2^	Mild bleeding	Proliferative phase of DAD with CMV inclusion	initiation of high-dose steroid and antivirus treatment	Rehab
3	62/F	IGT	Severe	PCV (PC 24 cmH_2_O, F_i_O_2_ 70%, PEEP 10 cmH_2_O)	RP-EBUS	2.4 mm/4 s	4/9, 12, 16, 25 mm^2^	Mild bleeding	Foreign body granulomas	initiation of high-dose steroid and a determination of the aspiration etiology	Rehab
4	65/F	CA	Severe	PCV (PC 20 cmH_2_O, F_i_O_2_ 100%, PEEP 8 cmH_2_O)	RP-EBUS	2.4 mm/4 s	4/9, 12, 16, 25 mm^2^	Severe bleeding	Fibrotic NSIP	steroid discontinuation and transitioned to palliative measures	Died
5	31/M		moderate	HFNC (F_i_O_2_ 40%, gas flow rate 60 L/min)	RP-EBUS and CBCT	2.4 mm/4 s	4/9, 15, 24, 42 mm^2^	Mild bleeding	COP	initiation of high-dose steroid	Rehab

*M* man; *F* woman; *MS* multiple sclerosis; *NS* nephrotic syndrome; *IGT* impaired glucose tolerance; *CA* lung adenocarcinoma; *PCV* pressure control ventilation; *PEEP* positive end expiratory pressure; *PC* pressure control above PEEP; *ECMO* extracorporeal membrane oxygenation; *RP-EBUS* radial probe endobronchial ultrasound; *CBCT* cone beam computed tomography; *DAD* diffuse alveolar damage; *HFNC* high-flow nasal cannula oxygen therapy; *CMV* cytomegalovirus; *NSIP* non-specific interstitial pneumonia; *COP* cryptogenic organized pneumonia; *Rehab* rehabilitation

### TBLC procedure

**Fig. 2:**
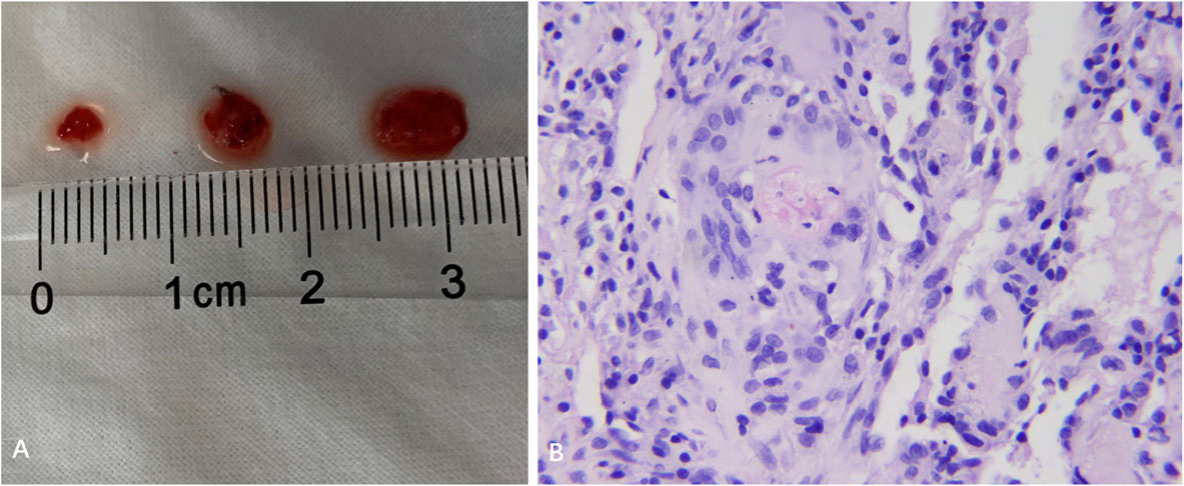
Specimens and histology obtained by transbronchial lung cryobiopsy for acute respiratory distress syndrome. **a** Gross specimens and their sizes. **b** Histologic diagnosis of foreign body granulomas in patient 3

All TBLC procedures were successfully performed using a 2.4 mm cryoprobe (freeze time: 4 s). A mean of 3.2 samples (range: 2–4 samples) were obtained from one (1 patient) or two (4 patients) lung segments. The samples had a mean size of 27.1 mm^2^ (surface area) and were deemed satisfactory for use in histopathology and tissue culture (Fig. 2). The patient with moderate ARDS successfully recovered from general anaesthesia and was placed on HFNC after TBLC. None of the patients experienced significant oxygenation state changes before or after the procedure. No patient developed pneumothorax but two patients suffered massive bleeding, which ceased after 2 and 3 h, respectively, in response to tamponades using bronchial blockers. There was no incident of haemodynamic instability. Three other patients had mild bleeding. There were no procedure-related deaths. The bronchial balloon blockers in patients with ECMO were removed after confirming no active bleeding following the reinitiation of UFH.

### Outcomes

Histopathological diagnosis in the five patients included a fibrotic phase of DAD with underlying infection (*n* = 1), proliferative phase of DAD with cytomegalovirus inclusions (*n* = 1), foreign body granulomas (*n* = 1), fibrotic non-specific interstitial pneumonia (*n* = 1) and cryptogenic organizing pneumonia (COP) (*n* = 1). One patient had an *Acinetobacter baumanii* infection, confirmed by tissue culture. Management changes (such as those in antibiotic and steroid usage (Table 1)) were made in all patients. Three patients improved and subsequently underwent rehabilitation treatments. Two patients died: one due to pulmonary embolism and the other because of septic shock.

### Systematic review

**Fig. 3:**
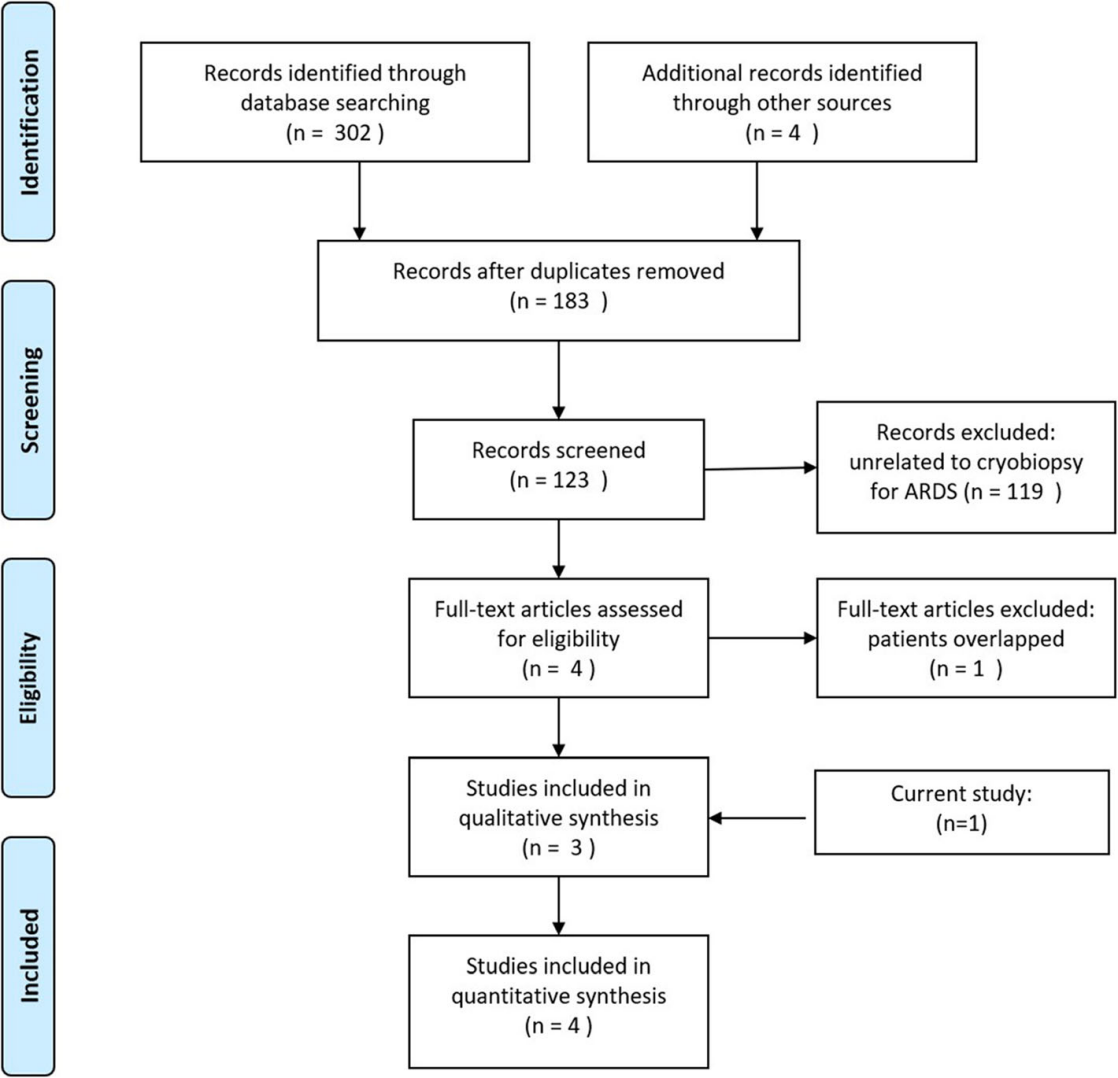
Flow diagram of the literature search and study selection process

Four studies (2 meeting abstracts, 1 published letter and 1 published paper) met the inclusion criteria [15–18] (Fig. 3). The patients in two studies [15, 18] overlapped such that three previous investigations of 20 patients with nonresolving ARDS who underwent TBLC were included [15–17]. Thus, together with our five patients, a total of 25 patients who met the criteria of the Berlin definition of ARDS were included in our larger study. The characteristics of these four studies are summarized in Table 2. All studies were retrospective case series. In the study of Dincer et al. [15], the five patients with ARDS were on MV and a mean of five specimens were obtained from each patient. A specific histopathological diagnosis was made and contributed to management changes in all patients. The pathological pattern included two cases of DAD and three alternative diagnoses. There were no procedure-related complications. Four of the five patients survived. Cooley et al. [16] reviewed 11 cases of ARDS in patients under MV support. The diagnostic yield of TBLC was 82% (9/11), including four cases of DAD. TBLC resulted in management changes in eight patients. Complications included pneumothorax (*n* = 4), persistent air leak (*n* = 2), and significant bleeding (*n* = 1). Six patients survived. Las Heras et al. [17] reported on four ARDS patients, and a specific histopathological diagnosis was made in each case, including DAD (*n* = 3) and another pattern (*n* = 1). TBLC contributed to management changes in two patients. There were no incidents of pneumothorax, significant bleeding or procedure-related death. The survival outcome was not available.

**Table 2:** Characteristics of the included studies with nonresolving acute respiratory distress syndrome (ARDS) patients undergoing transbronchial lung cryobiopsy (TBLC)

Study / year	Number of patients	Ventilation support	Final pathology diagnosis	Management changes	Complications	Survivals
Dincer et al./2018	5	MV	5/5 (2 DAD, 2 OP and 1 IPS)	5/5	None	4/5
Cooley et al./2019	11	MV	9/11 (4DAD, 2COP and 3 others)	8/11	4 pneumothorax, 2 persistent air leak, 1 significant bleeding	6/11
Las Heras et al./2019	4	MV	4/4 (3 DAD and 1 other)	2/4	None	NA
Current study	5	MV and ECMO	5/5 (2 DAD and 3 others)	5/5	2 significant bleeding	3/5
Summary	25	–	23/25 (11/25 DAD)	20/25	4/25 pneumothorax, 3/25 significant bleeding, 2/25 persistent air leak	13/21

*MV* mechanical ventilation; *ECMO* extracorporeal membrane oxygenation; *DAD* diffuse alveolar damage; *IPS* idiopathic pneumonia syndrome; *OP* organized pneumonia; *COP* cryptogenic organizing pneumonia; *NA* not available

According to the summary proportions from these four studies, the diagnostic yield of TBLC for nonresolving ARDS was 92% (23/25). DAD was diagnosed in 44% (11/25) of patients; the remainder had other diagnoses. TBLC contributed to management changes in 80% (20/25) of patients. Procedure-related complications included pneumothorax (16%, 4/25), significant bleeding (12%, 3/25) and persistent air leak (8%, 2/25). The rate of significant bleeding was 8.7% (2/23) in patients without ECMO. There were no procedure-related deaths. The follow-up survival rate was 61.9% (13/21).

## Discussion

We assessed the safety and value of TBLC for ARDS in five patients with nonresolving ARDS at our hospital and then in a larger study that also included all patients from previous studies.

TBLC is most often used in patients with DPLD. A recent study [13] indicated that the diagnostic yield of TBLC is comparable to that of SLB, with a high level of diagnostic agreement between the two procedures. Furthermore, the feasibility and diagnostic value of TBLC has been demonstrated in patients after lung transplantation [19] and in those with peripheral pulmonary lesions [20]. However, the safety and potential value of TBLC in ARDS has not been established. A literature search of this topic was conducted and resulted in the identification of only three independent studies with a total of 20 patients [15–17]. Our combined analyses suggested that complications of TBLC in ARDS are acceptable and similar to those reported for TBLC used in DPLD during the stable stage [12]. The overall complication rate of SLB is approximately 30%, and most of these cases (71%) are persistent air leaks [21]; this rate of complications is significantly higher than that for TBLC (8%). Persistent air leak will increase the difficulty of management in ARDS patients with MV. And it may lead to increased duration of MV, hospital length of stay and hospital mortality [22]. Another advantage of TBLC over SLB is that patient and ventilation conditions may be easier to manage, and the requirement for surgeon skill may be lower. Finally, the cost of TBLC is much lower than that of SLB [23]. In China, the overall cost of TBLC is approximately 10,000 yuan compared to approximately 50,000 yuan for SLB.

However, ARDS is defined only according to clinical and radiological criteria without consideration of the pathological findings, which are known to be heterogeneous. Identifying specific pathological patterns is an important determinant of treatment. Gerard et al. [6] reported that 57% (29/51) of patients who underwent SLB had pathologies other than DAD. Moreover, 37% (19/51) of the patients had a steroid-sensitive pathologic disease pattern (OP, acute interstitial pneumonia, acute exacerbation of usual interstitial pneumonia, eosinophilic pneumonia, pneumocystis pneumonia, alveolar haemorrhage and amiodarone toxicity); these patients had significantly better outcomes than patients with steroid-resistant pathologies (in-hospital mortality rate: 37% vs. 65%; 180-day mortality rate: 37% vs. 75%). In our study, based on the combined data of reported cases, the summary diagnostic yield of TBLC was 92, and 56% of patients had a diagnosis other than DAD. Changes in management occurred after multidisciplinary discussions, based on the clinical condition, laboratory data, BAL results of the patient, and the pathological pattern of TBLC in our institute. TBLC led to management changes in 80% of patients after including reported cases, which was similar to SLB [21]. These results indicate that TBLC may be an alternative biopsy method to SLB for patients with nonresolving ARDS.

Patients with ARDS typically have complex underlying conditions that increase the difficulty and risk of TBLC. ECMO is an important life support method for patients with severe ARDS. Almost all patients receive UFH to prevent thrombus formation and maintain normal ECMO operation, although the risk for procedure-related bleeding is higher. The use of TBLC in ARDS patients on ECMO has not previously been described. However, our study included two patients with severe ARDS who were on ECMO support in whom TBLC was successfully performed. One suffered from procedure-related massive bleeding, which was successfully stopped by 3 h of continuous occlusion using bronchial blockers. Thus, in patients with ECMO, TBLC should be performed after controlling for the bleeding risk and thrombogenesis, and bronchial blockers should be placed prophylactically. The rate of significant bleeding was 8.7% (2/23) in patients without ECMO.

The safety of TBLC depends to a large extent on the location of the cryoprobe [24]. A distance of < 1 cm to the pleura is associated with a significantly higher risk for pneumothorax, and a biopsy obtained too proximal to the middle third of the lung increases the risk for severe bleeding. RP-EBUS can identify diffuse lung lesions and their surrounding vessels, and encouraging results have been obtained using RP-EBUS-guided TBLC in patients with ILD [25, 26]. Our study is the first to describe the use of RP-EBUS-guided TBLC in ARDS. Four patients with severe ARDS underwent ICU-bedside TBLC guided only by RP-EBUS. The patient with moderate ARDS underwent TBLC in a hybrid OR with the combined guidance of CBCT and RP-EBUS. None of the patients developed pneumothorax, but two had massive bleeding. Thus, the value of RP-EBUS guidance requires further investigations in larger populations.

By including all patients from current and previous studies, our study became the largest case series to preliminarily assess the safety and value of TBLC in ARDS. However, it had several limitations. First, it was a retrospective study, although the results support the need for a prospective controlled study with a larger population. Second, the procedure protocols were not similar between studies. Further investigations should be performed based on a standard TBLC protocol for patients with ARDS. Third, the results were limited by the small sample size, and strong support for the routine use of TBLC in patients with nonresolving ARDS is still lacking. TBLC should be performed after multidisciplinary discussion with consideration of the clinical condition, laboratory data of the patient, and other noninvasive methods, such as endotracheal aspirates which can be used to investigate lower airway inflammation [27]. The cost/benefit ratio should also be considered.

## Conclusions

The complications of TBLC in selected patients with nonresolving ARDS may be acceptable. The procedure may have a high diagnostic yield and can lead to a re-evaluation of the diagnosis as well as changes in patient management. Further investigations with a prospective design and a larger number of patients are required to confirm the value of TBLC for nonresolving ARDS.

## Data Availability

The datasets used and analysed during the current study are available from the corresponding author on reasonable request.

## Abbreviations

ARDS: Acute respiratory distress syndrome
ILD: Interstitial lung disease
SLB: Surgical lung biopsy
ETT: Endotracheal tube
OR: Operating room
TBLB: Transbronchial lung biopsy
TBLC: Transbronchial lung cryobiopsy
RP-EBUS: Radial probe endobronchial ultrasound
ACT: Activated clotting time of whole blood
UFH: Unfractionated heparin
PCV: Pressure control ventilation
PEEP: Positive end expiratory pressure
PC: Pressure control above PEEP
ECMO: Extracorporeal membrane oxygenation
CBCT: Cone beam computed tomography
DAD: Diffuse alveolar damage
HFNC: High-flow nasal cannula oxygen therapy
CMV: Cytomegalovirus
NSIP: Non-specific interstitial pneumonia
COP: Cryptogenic organized pneumonia
COP: Cryptogenic organizing pneumonia
